# Ca^2+^ binding induced sequential allosteric activation of sortase A: An example for ion-triggered conformational selection

**DOI:** 10.1371/journal.pone.0205057

**Published:** 2018-10-15

**Authors:** Ilke Ugur, Martin Schatte, Antoine Marion, Manuel Glaser, Mara Boenitz-Dulat, Iris Antes

**Affiliations:** 1 Center for Integrated Protein Science Munich at the TUM School of Life Sciences, Technische Universität München, Freising, Germany; 2 Enzymes and Protein Technologies, Roche GmbH, Penzberg, Germany; Russian Academy of Medical Sciences, RUSSIAN FEDERATION

## Abstract

The allosteric activation of the intrinsically disordered enzyme *Staphylococcus aureus* sortase A is initiated via binding of a Ca^2+^ ion. Although Ca^2+^ binding was shown to initiate structural changes inducing disorder-to-order transitions, the details of the allosteric activation mechanism remain elusive. We performed long-term molecular dynamics simulations of sortase A without (3 simulations of 1.6 μs) and with bound Ca^2+^ (simulations of 1.6 μs, 1.8 μs, and 2.5 μs). Our results show that Ca^2+^ binding causes not only ordering of the disordered β6/β7 loop of the protein, but also modulates hinge motions in the dynamic β7/β8 loop, which is important for the catalytic activity of the enzyme. Cation binding triggers signal transmission from the Ca^2+^ binding site to the dynamic β7/β8 loop via the repetitive folding/unfolding of short helical stretches of the disordered β6/β7 loop. These correlated structural rearrangements lead to several distinct conformational states of the binding groove, which show optimal binding features for the sorting signal motif and feature binding energies up to 20 kcal/mol more favorable than observed for the sortase A without Ca^2+^. The presented results indicate a highly correlated, conformational selection-based activation mechanism of the enzyme triggered by cation binding. They also demonstrate the importance of the dynamics of intrinsically disordered regions for allosteric regulation.

## Introduction

Sortase transpeptidases are prokaryotic enzymes responsible for covalently attaching specific surface proteins to the bacterial cell wall envelope, which are critical for the virulence of the bacteria and also for their survival within the host [1]. The so bound proteins can be e.g. piles [2], nutrition binding proteins [3], or virulence factors like MSCRAMMs (Microbial Surface Components Recognizing Adhesive Matrix Molecules) [4], which mediate the attachment and interaction of bacteria with the host tissue. Examples are Clumping factor A (ClfA) [5], Collagen-Binding Adhesin (Acb) [6], or Protein A [7]. As all of these proteins play a major role in the virulence of e.g. *Staphylococcus aureus* [8, 9], they could be targeted simultaneously by the inhibition of sortases for e.g. the development of new antibiotic drugs. These proteins are recognized by sortases via a highly conserved amino acid sequence (LPXTG, where X is any amino acid). Binding of the LPXTG motif (also known as the sorting signal) to the active site of sortases triggers a series of biochemical reactions resulting in the site-specific adhesion of the surface proteins to the cell wall. Due to their key role in pathogen-host interaction, and their high efficiency in catalyzing a number of biomolecular blending reactions, sortase peptidases have been subject to both pharmaceutical [10–12] as well as enzyme engineering research.[13–21]. Recently, the sortase family has been subject to various molecular modeling studies [22–29] due to the enzymes’ allosteric regulation involving significant dynamical changes and their complex multistep enzymatic reaction mechanism.

**Fig 1:**
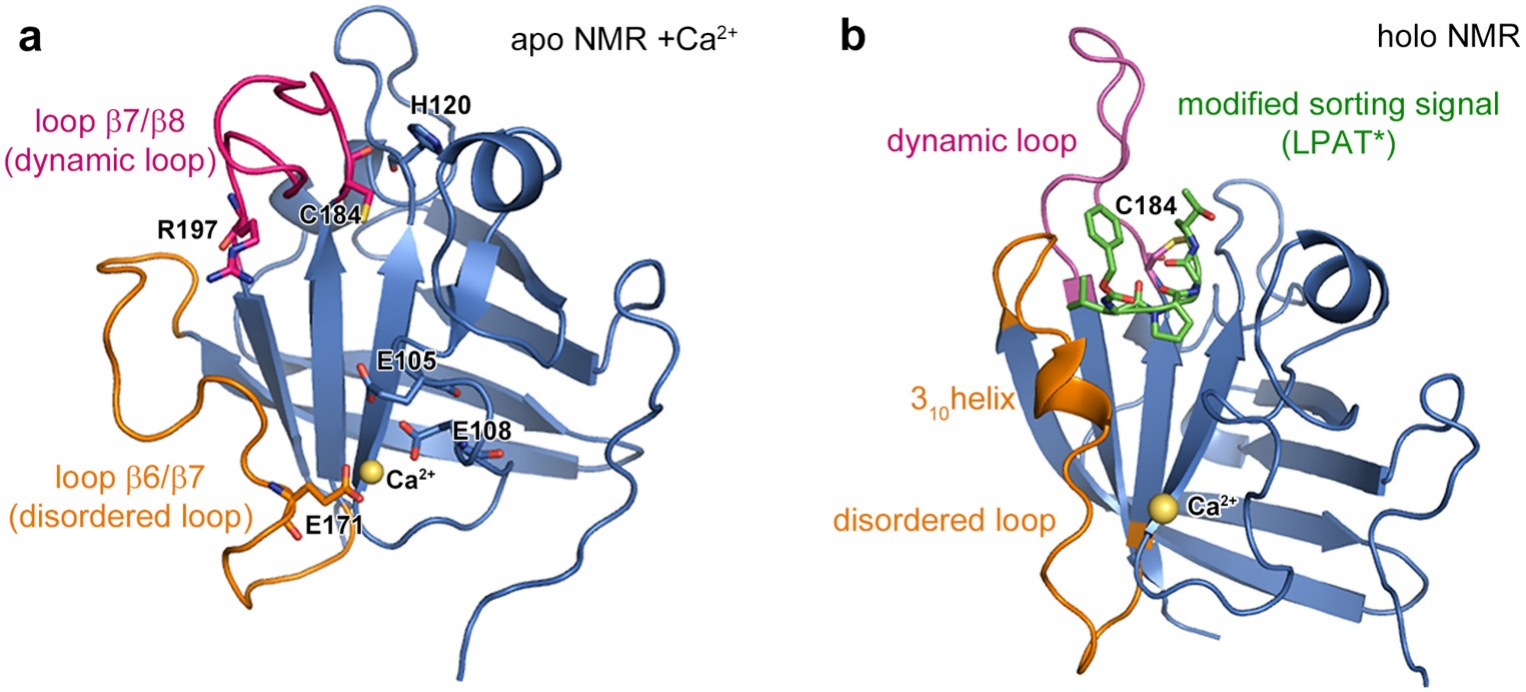
NMR-based structures of apo- and holo-SrtA. a) The structure of apo-SrtA (PDB: 1IJA [36]) is shown in cartoon representation. A Ca^2+^ ion is artificially added to the figure to show the initial structure used for the StrA-Ca simulations. The catalytically crucial amino acids and Ca^2+^ binding residues are shown in stick representation. Dynamic and disordered loops are colored in magenta and orange, respectively. b) Holo-structure of SrtA with bound Ca^2+^ and a covalently attached modified sorting signal (PDB: 2KID [33] LPAT*, green stick representation).

Three amino acids are known to be crucial to the catalytic activity of sortases: C184, H120, and R197 (Fig 1). Among these three residues, only the role of C184 is understood well, particularly for the *S*. *aureus* sortase A (Sa-SrtA), which is the best-studied member of the sortase family. The sorting signal covalently attaches to the sortase enzyme via bond formation between the side chain sulfur atom of C184 and the carbonyl carbon of the threonine residue in the LPXTG motif (sorting signal). This bond formation is the initial step of the transpeptidation reaction, leading to release of the terminal glycine residue. The thioacyl enzyme-substrate linkage is subsequently broken by the nucleophilic attack of a so-called cross-bridge peptide (consisting of a chain of 3–5 glycine residues) of a cell wall peptidoglycan. With the release of the sortase enzyme for the next enzymatic cycle, the surface protein is anchored to the cell wall. In this multistep reaction mechanism, H120 is suggested to act as a general base and to shuttle a H^+^ from one intermediate to another thus easing the release of the glycine [30, 31]. The most recently suggested roles for R197 are: stabilization of either the sorting signal [25, 32–34] or the negatively charged intermediates [30–32], or acting as a general base [35].

The three dimensional structure and dynamics of Sa-SrtA was profoundly studied to gain deeper insights into the biomolecular blending reaction mechanism. Structural studies showed that the Sa-SrtA skeleton consists of a stable eight-stranded β–barrel fold [36, 37]. Among several loops connecting these β–barrels, two of them (β6/β7 and β7/β8) were reported to be directly correlated to either sorting signal binding or to the catalytic activity (Fig 1). One of these loops -β7/β8- includes two of the catalytically crucial residues: C184 and R197. The sorting signal was suggested to bind to a groove, which is located at the intersection between the upper and lower regions of the β6/β7, and β7/β8 loops, respectively.

NMR studies provided crucial information on the conformational variety of these loops and on their engagement with the sorting signal [33]. These studies were performed on apo- and peptide-bound structures in which the LPXTG motif was modified and covalently attached to the Sa-SrtA, with LPAT bound to C184 of Sa-SrtA via a disulphide bond (Fig 1b). According to this study, the β7/β8 loop has two main rigid conformations: closed and open in the absence or presence of the sorting signal, respectively (Fig 1a and 1b). The β6/β7 loop has a predominantly disordered structure and can occupy several distinct conformations in the absence of the sorting signal. Binding of the sorting signal yields significant structural changes in the β6/β7 loop (Fig 1b), such as formation of a 3_10_ helix, and eventually the immobilization of the loop. Due to these structural features, the two loops are designated as disordered (β6/β7) and dynamic (β7/β8), and they will be referred to as such in the proceedings of this manuscript.

In the same NMR study [33], the authors showed that these features of the two active site loops are unique for Sa-SrtA. Two other investigated enzymes (Sp-SrtA and *Streptococcus pneumoniae* SrtC-1) contain a dynamic loop shorter than that of Sa-SrtA, which, in contrary to the Sa-SrtA loop, remains in an open conformation in the apo-state and in addition, the 3_10_ helical region of the β6/β7 loop is observed in the apo- as well as the holo-state for both of these enzymes (Sp-SrtA and *S*. *pneumoniae* SrtC-1), while in the case of Sa-SrtA, the latter only forms in the peptide-bound state (Fig 1).

Next to the conformational and structural differences in the two active site loops, Sa-SrtA has another crucial unique feature within the sortase family: namely the dependence of its catalytic activity on the binding of Ca^2+^ [36]. The Ca^2+^ ion was found to bind close to the N-terminal region of the disordered loop, far from the catalytic site (Fig 1). Three glutamate residues stabilize the ion in the grove: E105, E108, and E171, the latter being located in the disordered loop. The distance between the ion and the substrate binding site is approximately 18 Å. An indirect activation mechanism was thus suggested: Ca^2+^ binding to SrtA stabilizes the disordered loop in its closed helical conformation, which subsequently facilitates substrate binding [33, 36].

Similar ion binding and subsequent acceleration of the enzyme reaction has never been observed in any of the other sortase enzymes. In Sp-SrtA one of the negatively charged glutamates (E108) contributing to Ca^2+^ binding in Sa-SrtA is replaced by a positively charged lysine [33], thus effectively prohibiting Ca^2+^ binding due to charge repulsion. Inspired by this structural difference in Sp-SrtA, site specific mutations were performed in Sa-SrtA to investigate if cation independent functioning is possible [38]. Mutation of one of the Ca^2+^ binding glutamates (E105 or E108) led to an inactivation of the enzyme, whereas double mutations (E105K/E108A or E105K/E108Q) interestingly resulted in Ca^2+^ independent activity of the enzyme. However, in the latter case, the kinetics was slower compared to the Ca^2+^ dependent wild type Sa-SrtA. [38]

Due to the observation that the apo-structure occupies a larger conformational space compared to the substrate bound state, Sa-SrtA is classified as an intrinsically disordered protein (IDP). The disorder-to-order transition of the disordered (β6/β7) loop was suggested to be allosterically regulated by Ca^2+^ binding as the efficiency of this enzyme is increased upon Ca^2+^ binding. To reveal the mechanism of the disordered-to-order transition in Sa-SrtA, molecular dynamics simulations were previously performed for the apo- and Ca^2+^/peptide bound enzyme [22–24]. Moritsugu *et al*. used the multiscale enhanced sampling (MSES) simulation method to simulate four different states (apo, Ca^2+^ bound, peptide bound, Ca^2+^ + peptide bound) for 50–100 ns [22]. The authors suggested that the upper and lower regions of the disordered loop cooperatively stabilize the ligand bound conformation once both ligands (Ca^2+^ + peptide) are bound. They also observed positive and negative correlations between the motions of the dynamic loop and the upper and lower regions of the disordered loop, respectively. Pang *et al*. performed simulations using the replica exchange with solute tempering method (REST), which showed ordering of the disordered loop upon Ca^2+^ binding [24]. As the two studies used different sampling methods, simulation times, and force fields, they eventually sampled different regions of the conformational spaces of the apo- and the holo-Sa-SrtA. However, despite the fundamental differences in their methodologies, both studies indicate that there exists a correlation between the two loops and, without performing further calculations, proposed a possible increase in the binding affinity of the sorting signal upon Ca^2+^ binding.

In the present study, we performed long-term molecular dynamics simulations of the apo- and Ca^2+^-bound form of Sa-SrtA to investigate the dynamical alterations caused by Ca^2+^ binding and to gain a more detailed understanding regarding the allosteric activation mechanism and the role of Ca^2+^ on substrate binding. Using structurally distinct conformations obtained from our long-term simulations, we calculated the differences in the binding affinity of the sorting signal peptide upon Ca^2+^ binding. Our computational observations are supported by differential scanning calorimetry (DSC) measurements of Sa-SrtA in the presence and absence of Ca^2+^.

The results from this study provide new insights into the complex sortase reaction mechanism, which can guide future sortase engineering campaigns as well as the development of improved sortase inhibitors, such as new antibiotic drugs. The latter can help to fight the growing numbers of pathogens showing multiple-drug resistance, one of the major challenges for mankind [39].

## Materials and methods

### Preparation of the structures

Both simulated systems, SrtA-noCa and SrtA-Ca, were built based on the first model of the NMR apo-structure of SrtA (PDB: 1IJA). To prepare the SrtA-Ca model, a Ca^2+^ ion was placed in the space between the residues E105, E108, and E171, as those residues were shown to bind to Ca^2+^ [36] (see Fig 1a). The systems were solvated in a rectangular water box using tleap module of the Amber14/AmberTools15 [40] program package, by applying a 12 Å buffer region around protein atoms. The solvated systems were neutralized to yield models consisting of ~30,000 atoms.

### Molecular dynamic simulations

All simulations were performed using the ff03[41] and TIP3P[42] force field parameters for the solute and solvent, respectively. Periodic boundary conditions were applied to each system and long-range electrostatic interactions were calculated using the particle mesh Ewald method [43]. A non-bonded cutoff of 12 Å and a time step of 1 fs were used during all simulations (heat up, equilibration and production). Temperature and pressure were controlled using a Langevin thermostat and Berendsen barostat, respectively, with default conditions as implemented in Amber14. Prior to minimization of the models, the density of the systems was adjusted to 1 g/cm^3^ using an in-house python script. The hydrogen and the heavy atoms were minimized consecutively using the SANDER module of Amber14. The systems were heated up to 300 K in the NVT ensemble, in a stepwise fashion as performed in our previous work applying a 3 kcal.mol^-1^.A^-2^ force constant first on all heavy protein atoms (0-50K) and later on all backbone atoms (50-200K), respectively [44, 45]. The SHAKE algorithm was used to constraint all bonds involving hydrogen atoms [46]. Based on the heated-up structure at 300K we performed three independent simulations of 1600 ns each for both systems, SrtA-noCa and SrtA-Ca, starting with randomized velocities. The most promising of the SrtA-Ca simulations were extended to 1800 and 2500 ns. These simulations were performed in the NPT ensemble, using the cuda-enabled graphics processing units (GPUS) version of the pmemd module of Amber14 [47, 48].

### Molecular docking calculations

The sorting signal sequence was chosen as LPATG. The N and C terminal ends were capped with acetyl and N-methyl amide, respectively. Twelve different receptor conformations were chosen for the molecular docking calculations. These structures correspond to representative structures observed during the long-term molecular dynamics simulations (7 for SrtA-Ca and 5 for SrtA-noCa, see Results section for details). Docking of the sorting signal was performed into each of these structures using the DynaDock approach of our in-house modeling program DynaCell [49]. The DynaDock approach consists of two steps; first, conformational sampling of the ligand in the binding site, and second, a molecular dynamics based energy refinement step of selected poses using a softcore-based potential and specialized MD algorithm (Optimized Potential Molecular Dynamics, OPMD). During the conformational sampling, translational and rotational movements of the whole ligand up to 2 Å and 30° were performed and all torsional degrees of freedom of the peptide were treated as flexible using a rotation angle of 30°. An overlap between ligand and receptor atoms of up to 80% of their Van der Waals radii was allowed (see ref [49] for further explanation). The sampled ligand poses were clustered by hierarchical clustering as implemented in cpptraj (heavy atoms of the sorting signal only) using default settings and a RMSD of 4 Å. 200 representative structures belonging to the highest populated clusters, bearing the highest interaction energy and in which the ligand was located within 10 Å radius of the active site (Cys184) were chosen for OPMD refinement. For these structures OPMD simulations were performed for 20 ps to eliminate any Van der Waals overlap using backbone restraints with a force constant of 100 kJ.mol^−1^.nm^−2^ (see ref [49]). All successfully refined structures (overlap = 0%) were further clustered same as above, but using a RMSD of 2 Å and poses belonging to highly populated clusters and featuring very favorable interaction energies were inspected visually. The 2–3 best poses with respect to their structural resemblance to the NMR holo structure (PDB: 2KID)[33] and their position in the active site (i.e. the distance from Cys184 and the top of the disordered loop) were chosen for further investigation and minimized, heated-up, and equilibrated for 5ns (using the same protocol as described above). The most stable pose was chosen for MMGBSA binding free energy estimations.

### MMGBSA binding free energy estimations

As basis for the MMGBSA calculations three distinct simulations (starting with different velocities) were performed for each equilibrated structure for further 15 ns, using a time step of 1 fs and saving a total of 225,000 complex frames (3 x 75,000). The binding free energies were estimated using the Molecular Mechanics-Generalized Born Surface Area approach [50] by means of the MMGBSA.py module [51] of Amber14/AmberTools15 [40]. The contribution of the solvent was computed using the default Generalized Born Surface Area (GBSA) options of the script with a probe radius of 1.4 Å and the ‘mbondi2’ radii set [52]. The entropic contributions to the free energy of binding were not included in the calculation scheme, as it has been shown that such costly computation does not significantly improve the results [53–55]. Despite the fact that the entropic contributions were not added to the calculation, the binding energy terms are referred with the letter G for consistency with the published literature.

### Sortase expression and purification

Cloning, overexpression and purification of the wild type Sa-SrtAΔ59 and P94S variant was performed according to the method of Ton-That et al. [56]. In this study a pQE-80L vector and BL21 *Escherichia coli* cells were used for overnight expression in LB-media. For purification, the cells were lysed via French press and centrifuged. The supernatant was applied to a Ni-NTA resin and afterwards to a superdex 60 column. The final product was dialyzed against sortase buffer (50 mM Tris/HCl, 200 mM NaCl, 10 mM CaCl_2_, pH 7.5) or buffer without Ca^2+^ and EDTA (50 mM Tris/HCl, 200 mM NaCl, 5 mM EDTA, pH 7.5) followed by dialyzing against buffer without EDTA and Ca^2+^ (50 mM Tris/HCl, 200 mM NaCl, pH 7.5).

### Differential scanning calorimetry (DSC) measurements

The analysis was performed on a Malvern MicroCal instrument using buffer without SrtA as reference (50 mM Tris/HCl, 200 mM NaCl, pH 7.5) with and without 10 mM Ca^2+^. The capillary was heated from 20 to 90°C with a rate of 90°C/h. The differences in heat flow were monitored.

## Results and discussion

We performed three independent replica simulations for both of the systems, SrtA with and without calcium (named SrtA-Ca and SrtA-noCa, respectively), to ensure the solidity of the observed conformational changes. Analysis of the trajectories showed that the similar conformational changes occurred in all three replicas, the main difference being the simulation time between individual events. Thus, to obtain a comprehensive picture of the conformational dynamics of the simulated systems we chose the simulations with the most prominent conformational changes as reference simulations for further analysis.

**Fig 2:**
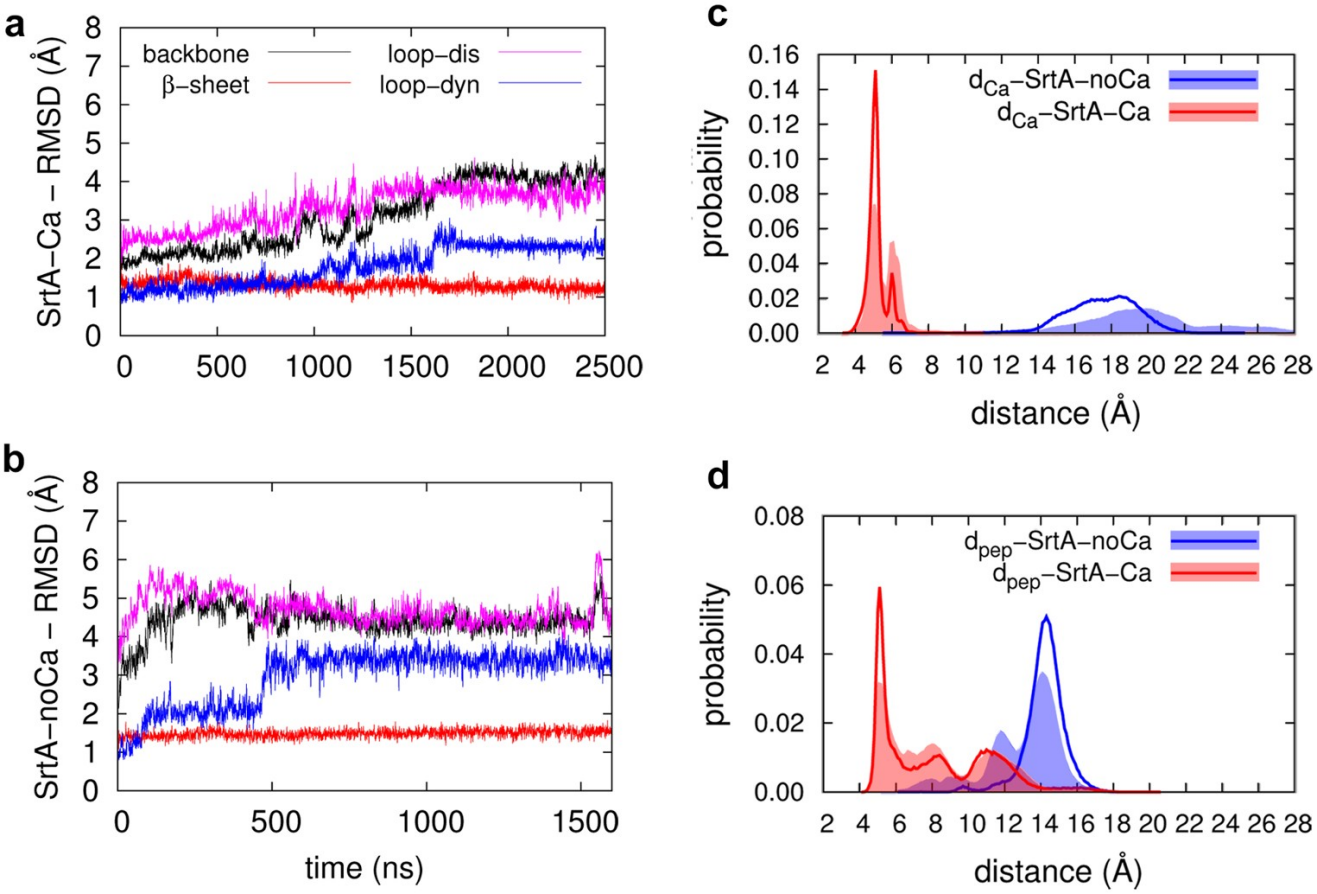
Atom-positional RMSD and probability distributions of key distances. a) and b) Atom-positional RMSD (Å) from the starting structures as observed during the two representative simulations of SrtA-Ca and SrtA-noCa, respectively. The black, red, magenta, and blue colors represent the deviations of the backbone atoms of the protein, residues assembling the eight-stranded β-barrel (β sheets), the disordered loop (loop-dis), and the dynamic loop (loop-dyn), respectively; c) and d) Probability distributions of the d_Ca_ and d_pep_ distances (Å) for the two representative simulations of SrtA-noCa (red line) and SrtA-Ca (blue line). Filled curves show the combined probability of the three replica simulations.

As our main goal was to investigate the long-term dynamics of sortase A after Ca^2+^ binding, we monitored the overall movement and the stability of the simulated systems by following the overall root mean square deviation (RMSD) of the protein backbone with respect to the experimental starting structure. In Fig 2a and 2b, the RMSD’s of the protein as well as the residues assembling the eight-stranded β-barrel and the disordered and dynamic loops are monitored individually (Fig 2a and 2b, red, magenta and blue lines, respectively). For both simulations, the RMSD of the stable eight-stranded β-barrel is nearly constant during the simulations with a small value of ~1.5 Å, indicating a stable and dependable simulation for both systems. During the SrtA-Ca simulation, the RMSD of the protein backbone gradually increases and stabilizes around 4 Å after ~1500 ns. The same trend can be observed for the two loops, whereas the RMSD values are larger for the disordered loop than for the dynamic loop (magenta and blue lines, respectively). This simultaneous increase and stabilization of the RMSD values of the loops point to a correlation between the respective motions of the two loops, which is further detailed in the following discussion. During the other two replica simulations of the SrtA-Ca system the same overall motions were observed, although RMSD-wise the total movement of the dynamic loop is found to be much smaller and the predominant contribution to the overall RMSD originates from the dynamics of the disordered loop (S1a and S1b Fig).

A similar correlation between the dynamics of the two loops can be observed for the representative simulation of the cation-free-structure (SrtA-noCa) (Fig 2b), with the dynamic and disordered loops reaching a steady state after 500 ns. In this case, however, a different motion pattern was observed during the other two replica simulations (S1c and S1d Fig) with the disordered loop showing large, uncorrelated movements, yielding RMSD values greater than 6 Å. This indicates that in the calcium free state the correlation between the two loops is very small.

Previously, Moritsugu *et al*. proposed two distance-based descriptors to measure the formation of the binding pockets of Ca^2+^ and the sorting signal, respectively [22]. First, the distance between the Cα atoms of P163 and R197 was chosen for the sorting signal pocket (d_pep_). Second, the minimum value of the distance between the Cδ atom of E171 and the other Ca^2+^ binding residues E105, E108 (Fig 1) was used for the Ca^2+^ binding pocket (d_ca_). Pang *et al*. used the same descriptors to compare the results of their REST simulations with this earlier study [24]. To evaluate and verify the convergence of our long-term MD simulations in comparison to these two earlier extensive studies, which use advanced sampling techniques, but shorter simulation times, we monitored the changes in d_pep_ and d_ca_ during our simulations. We plotted both their probability distributions obtained from one single continuous simulation (Fig 2c and 2d, solid lines) and a combination of all three replica simulations (filled curves).

It can clearly be observed that the probability distributions obtained from the analysis of all three replica simulations show the same pattern as the individual distributions of the representative simulations for both SrtA-Ca and SrtA-noCa (Fig 2c and 2d, filled curves), only the intensities of the curves are slightly affected. This demonstrates that all three replica simulations show very similar results with respect to the important structural features of the system. In the Ca^2+^ containing simulation (SrtA-Ca), d_ca_ is well defined as a narrow peak around 5 Å (red line, Fig 2c). If there is no Ca^2+^ (SrtA-noCa, blue line), the peak is broad and is located around 20 Å. These results are in general agreement with the previous data, except that in the REST study the largest peak is located at a slightly shorter distance (17 Å). The d_ca_ value is predominantly independent of the sampling method and the force field used: if Ca^2+^ is bound to the system, it bridges and stabilizes the two coordinating glutamate residues yielding a narrow distribution centered around a short distance. In the absence of the cation, the two negatively charged residues E171 and E105/E108 repel each other and move freely, resulting in a broad distribution and large intermolecular distance.

For d_pep_ we could observe three peaks in the SrtA-noCa simulation (Fig 2d, blue lines), one small (~ 8 Å) and two larger distributions (~12 and ~14 Å). If only the representative simulation is considered, the probability is predominantly distributed around 14 Å. The overall distribution is in accordance with the REST simulation study (two peaks at ~7 Å and 14 Å), whereas during the MSES simulations only one peak was detected (~8 Å). The distribution of d_pep_ during the SrtA-Ca simulations shows no significant differences between the representative simulation and the average values obtained from the three replicas: We detected three peaks at 5 Å, 8 Å, and 11 Å; the first peak being the sharpest and indicating a well-defined signal peptide binding site. Comparing this result to the previous simulation studies, there exist significant differences in peak detection depending on the simulation method applied. During the REST simulations one single d_pep_ value of 5 Å was detected, whereas the MSES study led to one d_pep_ value of 8 Å, respectively. Notably, our simulations include both peaks of the previous simulations and even show an additional peak at 11 Å. As such more comprehensive peak observations were previously suggested as a proof of a more extensive conformational sampling, we consider our long-term conventional MD simulations as being at least as comprehensive as the previous advanced sampling simulations.

**Fig 3:**
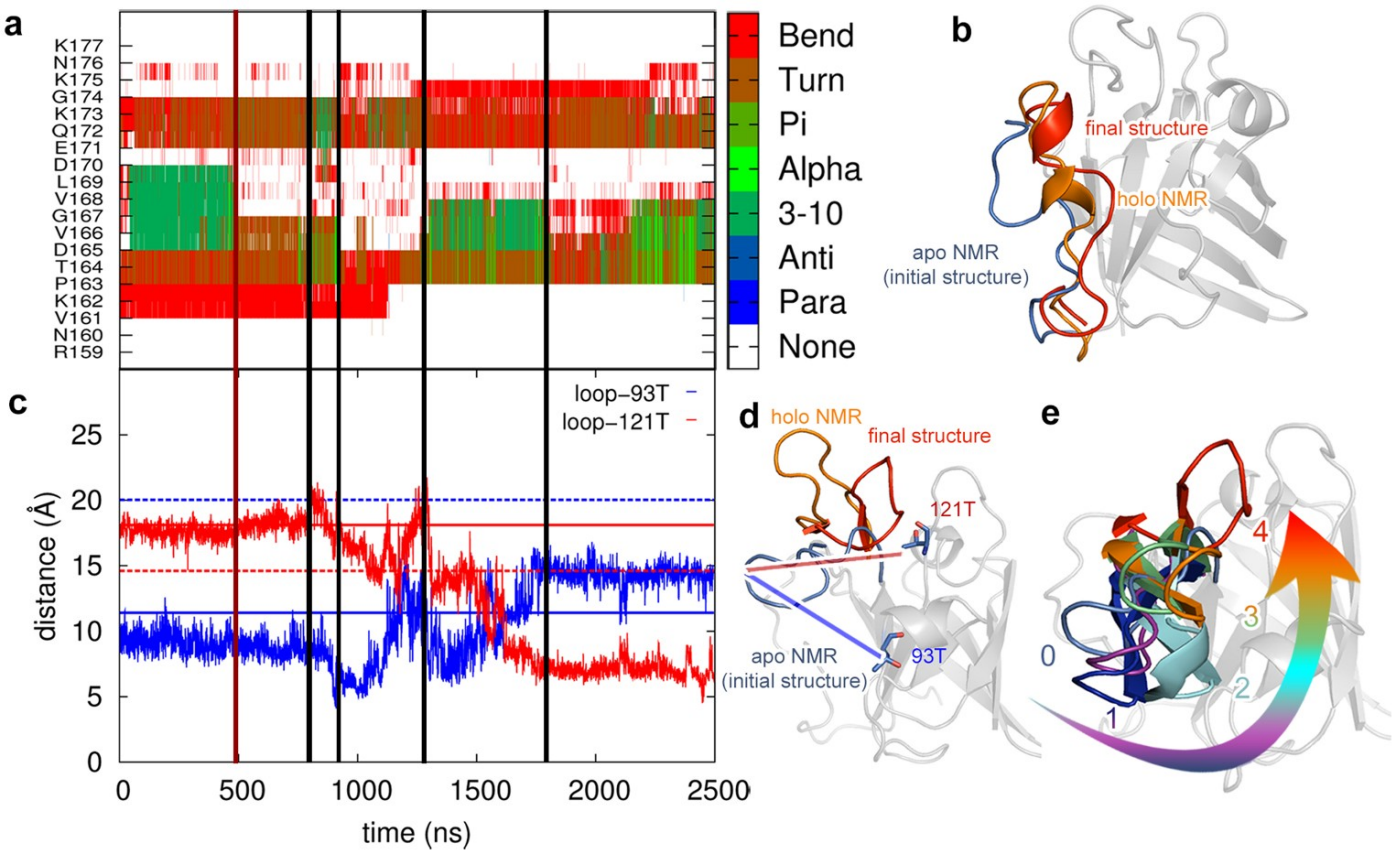
Structural changes in the disordered and the dynamic loops during the SrtA-Ca simulation. a) Changes in the secondary structure of the disordered loop. b) Representative structures of the disordered loop. c) Variations of the distances between the tip part of the loop (189E to 192G) and two reference amino acids 93T (blue lines) and 121T (red lines). Solid and dashed lines show the reference distances for the apo- and holo-NMR structures, respectively. d) Initial (blue), final (red), and holo NMR (orange) conformations of the dynamic loop. The residues used to follow loop motion are shown by stick representation (93T and 121T). e) Representative structures of the dynamic loop showing the direction of the opening.

Next, we analyzed the signal transmission pathway from the Ca^2+^ binding site to the substrate-binding groove. Previous NMR studies showed that Ca^2+^ and/or sorting signal binding introduce a stabilization of the disordered loop in SrtA, which is accompanied by the formation of a 3_10_ helix around residues V166-V168. Therefore, we followed the changes in the secondary structure of the disordered loop during the course of the simulations to investigate the 3_10_ helix formation for the SrtA-Ca system (Fig 3a). Formation of the 3_10_ helix occurs in the first 400 ns (green lines, Fig 3a) between the residues V165-L169, afterwards the loop relaxes up to 800 ns (red lines, Fig 3a). From this point to the end of the simulation (800–2500 ns) we observe folding (green) and unfolding (red, Fig 3a) of α helices at different ends of the loop including the residues: E171-G174, D165-V168, P163-V168. Overall, the disordered loop does not stabilize to one single conformation; but the formation/deformation of the helices in different regions of the loop generates several stable intermediate structures (Fig 3b). Similar observations can be made for the two replica simulations, in which the conformational switching occurs on a longer time scale, especially during the 3^rd^ simulation (S2 Fig). Analysing the SrtA-noCa simulation, also there folding and unfolding events of the 3_10_ helix can be detected, however, these events are predominantly located at the lower region of the loop (close to the Ca^2+^ binding site) and in contrary to the SrtA-Ca simulation no significant changes in the overall position of the upper region of the loop, close to the substrate binding site, can be observed (S3 Fig). In addition, the overall helical content differs much stronger between the individual replicas, indicating a significantly weaker tendency for helix formation (S4 Fig) than in the calcium bound state.

Next we analyzed the motion of the dynamic loop. For this we tracked the changes in distances between the tip region of the loop (E189 to G192) and two reference amino acids (T93 and T121, Fig 3d). These two reference amino acids were chosen considering three criteria: i) they remain stable during the simulation, ii) the distance from these residues to the tip of the loop differs more than 5 Å in the apo- and holo-NMR structures, and iii) they are located in the front (T93) and in the rear region (T121) of the protein with respect to the positioning of the loop (see the orientation in Fig 3d). The two distances are shown in Fig 3c (SrtA-Ca) and the reference values of the apo- and holo-structures are given in solid and dashed lines, respectively. During the first 800 ns, the distance between the loop and the reference residues is stable with a value close to that of the apo-structure (i.e., the initial structure of the simulation). Between 800–1800 ns, the red and blue lines decrease and increase respectively, this means the loop moves closer to the rear region forming several stable intermediate states (Fig 3e). From this point on, the position of the loop is stabilized (1800–2500 ns) in an open conformation. Overall, by comparing the beginning and the end of the simulation, it appears that the distances to the front (blue) and to the rare regions (red) of the protein interchange; revealing two stable and significantly distinct conformations (close and open) at the beginning and the end of the simulation (Fig 3d). In the final conformation, the dynamic loop is close to the open loop conformation as observed in the holo-NMR structure (orange colored, Fig 3d), but still not ‘fully opened’, since the final conformation (red colored, Fig 3d) still differs from the experimentally observed loop conformation and thus the two followed distances do not fully reached their reference values (dashed lines, Fig 3c). As no distinct movement can be observed for a long period at the end of the simulation, the question arises if the holo-structure is not reached due to the simulation time limit or if peptide binding is necessary to obtain the final holo-structure.

Combining our distance-based analysis with visual inspections of the trajectory, we could observe that the dynamic loop shows a counterclockwise rotational movement (Fig 3e, representative intermediates are colored differently and labeled with respect to their time occurrence in the trajectory 0 to 4). Overall, calcium binding triggers a hinge motion in the dynamic loop, which moves from the front to the rear region of the protein (approximately 10 Å more than in the apo-structure). In one of the replicas the loop briefly opens at the very end of the simulation and goes back to the open position (S2c Fig). The motion of the dynamic loop in the third replica is less significant (S2d Fig).

For the cation-free SrtA-noCa simulation, in one of the simulations the dynamic loop opens briefly and resembles the conformation of the holo-reference structure for a short period (150–400 ns; S3b Fig). For the rest of the simulation time, the loop retains its semi-open conformation and only shows a local twisting motion at its tip (S3c Fig). However, the overall loop fluctuations are larger compared to SrtA-Ca, indicating a less stable structure. This is also valid for the other two replica simulations (S4c and S4d Fig).

The comparison of the dynamical changes in these two loops in the presence of Ca^2+^, shows that the major alterations in the secondary structure of the disordered loop and the movement of the dynamic loop occur within the same time intervals (black horizontal lines in Fig 3a and 3c) revealing a strong correlation between these two loops, which is detected in all simulations performed (S2 Fig). Such a correlation has been previously explained simply by the steric impact of the N-terminal region of the disordered loop on the dynamic loop [24]. Our results allow a more detailed view of this dynamic interaction as they show that the opening of the dynamic loop involves several intermediate structures, which are correlated to consecutive changes in the secondary structure of the disordered loop that are triggered by Ca^2+^ binding.

As our analysis suggests that the intrinsic disordered loop becomes structurally ordered after Ca^2+^ binding, Ca^2+^ binding should stabilize the overall fold of Sa-SrtA and therefore lead to an increased thermal melting point of the protein. To provide experimental evidence for the stabilizing effect of Ca^2+^ on SrtA, DSC measurements were performed in the presence and absence of Ca^2+^. The melting point (T_m_) of Sa-SrtA with and without Ca^2+^ are 60.7 and 57.3 °C, respectively (S5 Fig). The increase of 3.4 °C in T_m_ with Ca^2+^ is highly significant and provides experimental evidence for our theoretical observation that calcium stabilizes the Sa-SrtA structure.

As it was previously suggested that Ca^2+^ binding not only stabilizes the protein, but also increases its affinity towards the signaling peptide, we performed peptide binding studies for selected protein conformations obtained from our simulation trajectories.

**Fig 4:**
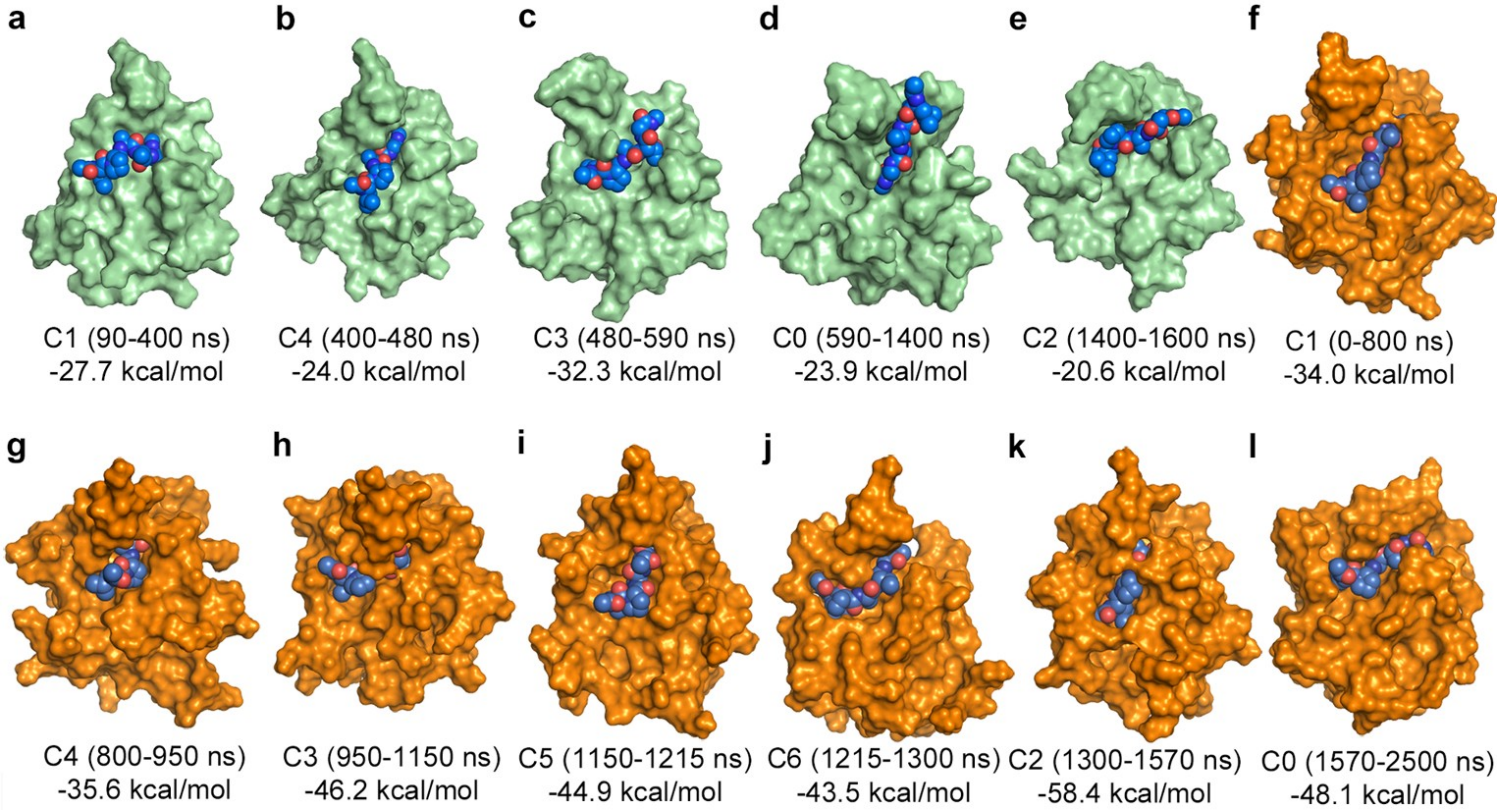
Structures and corresponding ΔG_bind_ values of the sorting signal for the final bound conformations after 5ns of equilibration. Results for the representative structures chosen for the molecular docking and MMGBSA calculations from the SrtA-noCa (a-e, green) and the SrtA-Ca (f-l, orange) simulations. The receptors are shown in surface, and the sorting signals in sphere representation.

Our representative 2500 ns simulation of SrtA-Ca yielded 8 distinct structural clusters with varying population rates within a 2 Å RMSD limit. We analyzed the distribution of these clusters along the trajectory and observed that they form stable intermediate conformations with limited interchanges and a minimum and maximum lifetime of 100 ns and 900 ns, respectively (S6 Fig). The short-lived cluster at the very beginning of the simulation is considered as the equilibration phase and therefore discarded from the discussion. Remarkably, the different clusters could be correlated to the concerted variations in the secondary structure of the disordered loop and the correlated changes in the dynamic loop discussed above, indicating that these motions generate distinct structural intermediate conformations during the Ca^2+^ binding induced apo- to holo-transition. Hence, we obtained seven stable intermediates, each of them bearing a distinguishable conformation of both of the two loops of interests (S7 Fig). The same analysis was performed for the representative simulation of SrtA-noCa as well, and the stable first five clusters were chosen for further analysis (S6b Fig). To investigate the influence of the above detailed conformational changes triggered by Ca^2+^ binding on the binding affinity of the sorting signal, we performed DynaDock molecular docking simulations of the sorting signal peptide into all twelve obtained structural clusters (seven for SrtA-Ca and five for SrtA-noCa) and calculated the binding affinities of the peptide using the MMGBSA approach. The calculated binding free energies (ΔG_bind_) and their components (ΔE_VdW_, ΔE_el_, ΔG_gb_, and ΔG_surf_) are given in Table 1 and the corresponding equilibrated structures of the sorting signal-bound SrtA complexes are shown in Fig 4. The binding affinities of the sorting signal bound to the SrtA-noCa structures are ~ 20 kcal/mol smaller than the affinities observed for the peptide bound to the calcium-bound sortase conformations (SrtA-Ca). Detailed structural analysis showed that the peptide binding groove is much more flexible in the SrtA-noCa structures compared to SrtA-Ca and thus the peptide is not able to adopt stable bound conformations (Fig 4a–4e). Comparing the results obtained for the different representative SrtA-Ca intermediates, it can be seen that the conformations occurring during the later stages of the simulation (after 950 ns) yield better ΔG_bind_ values (~ -45 kcal/mol) than the initially occurring conformations (~ -35 kcal/mol). This is consistent with the observation discussed in the previous section that the dynamic loop moves toward its open holo-like conformation during the end of the simulation (950–2500 ns, complexes C3, C5, C6, C2, and C0). This directly affects the overall binding site conformation, which then also resembles the final bound conformation much better. Practically, an increased interaction surface between the substrate peptide and the protein, leading to a more compact complex structure, can be observed (Fig 4). Therefore, the structural changes initiated by Ca^2+^ binding as detailed in the previous section, lead to an optimization of the binding groove conformation with respect to its binding complementarity to the sorting signal. The opening of the dynamic loop is particularly crucial in establishing these peptide binding-optimized binding groove conformations.

**Table 1:** MMGBSA-based binding affinities (ΔG_bind_) and their components (ΔE_VdW_, ΔE_el_, ΔG_gb_, ΔG_surf_) [kcal/mol] of the sorting signal peptide to the cluster representative conformations of SrtA as extracted from the simulations.

		ΔG_bind_	ΔE_VdW_	ΔE _el_	ΔG _gb_	ΔG _surf_
**SrtA-noCa**	C1 (90–400 ns)	-27.7	-38.7	-32.5	48.5	-5.0
	C4 (400–480 ns)	-24.0	-35.2	-21.5	37.3	-4.6
	C3 (480–590 ns)	-32.3	-41.5	-32.6	47.6	-5.8
	C0 (590-1400ns)	-23.9	-36.6	-15.3	33.1	-5.1
	C2 (1400–1600 ns)	-20.6	-31.7	-29.3	44.6	-4.2
**SrtA-Ca**	C1 (0–800 ns)	-34.0	-49.8	-25.3	47.5	-6.4
	C4 (800–950 ns)	-35.6	-55.0	-21.5	48.0	-7.2
	C3(950–1150 ns)	-46.2	-61.6	-35.1	58.4	-7.9
	C5(1150–1215 ns)	-44.9	-59.3	-41.5	63.2	-7.4
	C6(1215–1300 ns)	-43.5	-57.5	-42.2	63.8	-7.5
	C2(1300–1570 ns)	-58.4	-73.0	-38.8	57.5	-9.0
	C0(1570–2500 ns)	-48.1	-63.1	-39.5	62.6	-8.1

ΔE_VdW_ and ΔE_el_ are the Van der Waals and electrostatic energy contribution from molecular mechanics, and ΔG_gb_ and ΔG_surf_ are electrostatic and non-polar contribution to the solvation energy, respectively.

**Fig 5:**
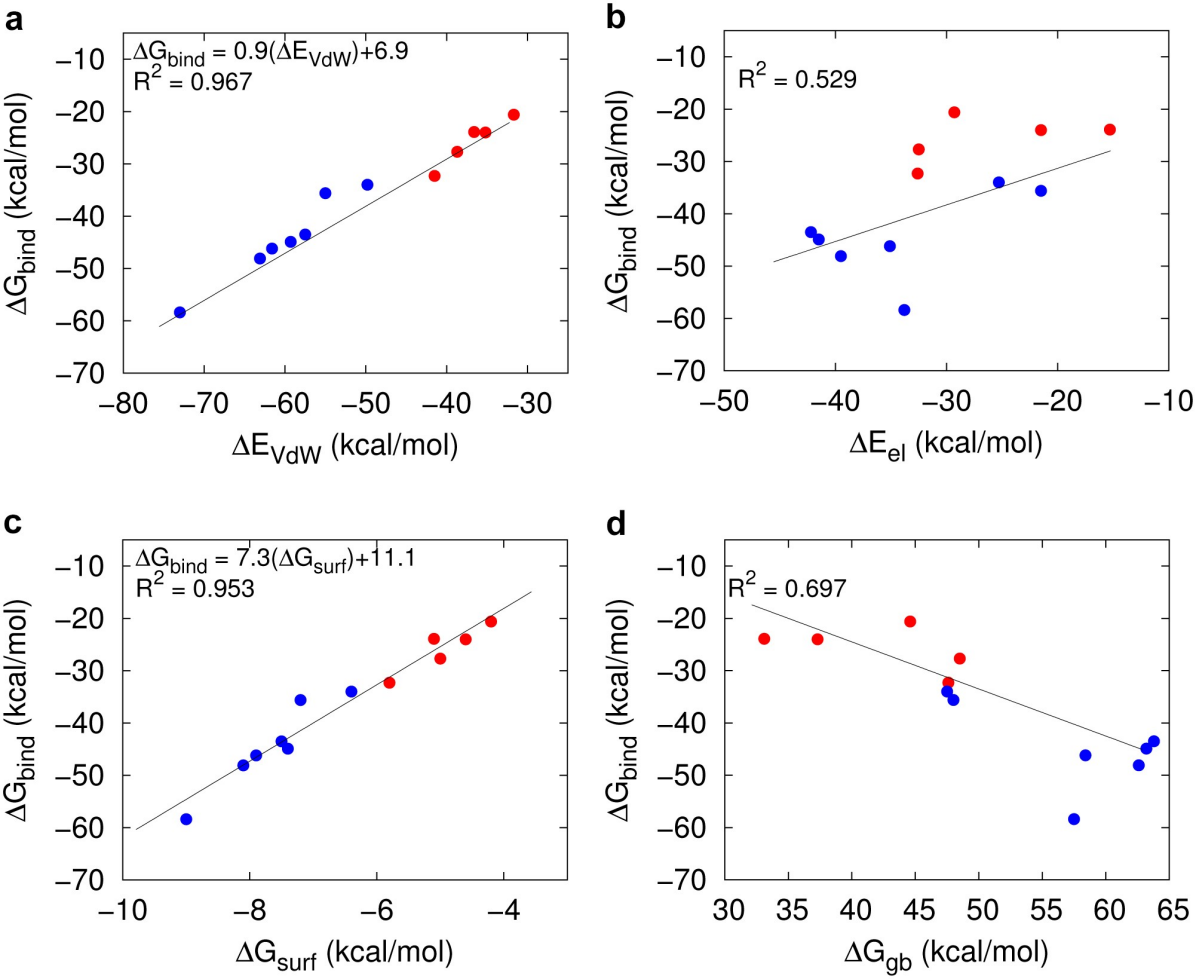
Correlation between total binding affinity (ΔG_bind_) and its individual components. a-b) Van der Waals (ΔE_VdW_) and electrostatic (ΔE_el_) contributions to the interaction energy, respectively. c-d) nonpolar (ΔG_surf_) and polar (ΔG_gb_) solvation free energies, respectively. The binding affinities and their components were calculated by MMGBSA for the cluster representatives extracted from the SrtA-Ca (blue points) and the SrtA-noCa (red points) simulations, respectively.

In the MMGBSA approach the binding free energy is approximated by the interaction energy between the ligand and the receptor, and the difference in solvation free energy upon binding. The electrostatic and non-electrostatic interactions between the ligand and the receptor are provided by ΔE_el_ and ΔE_VdW_, respectively. ΔG_gb_ and ΔG_surf_ are the electrostatic and non-polar contributions to the solvation free energy. We analyzed the correlation between ΔG_bind_ and its individual components (ΔE_VdW_, ΔE_el_, ΔG_gb_, and ΔG_surf_). All twelve conformations (SrtA-noCa and SrtA-Ca) were included into the calculations (Fig 5). To distinguish between the SrtA-Ca and the SrtA-noCa results, the corresponding values are plotted in blue for the former and in red for the latter. Among the four free energy components, a very strong linear correlation between ΔG_bind_ and the non-electrostatic contributions (ΔE_VdW_/ΔG_surf_) is found with linear correlation coefficients (R^2^) of 0.967 and 0.953, respectively (Fig 5a and 5c). The interrelation between ΔG_bind_ and the electrostatic components (ΔE_el_/ΔG_gb_) is much lower with R^2^ values of 0.529 and 0.697, respectively (Fig 5b and 5d). Such positive correlation between the buried nonpolar surface area and the binding free energy was previously shown for a number of peptidic ligands [57]. These remarkably strong correlations between ΔG_bind_ and ΔE_VdW_ (or ΔG_surf_) indicate that the binding of the sorting signal is mainly driven by hydrophobic interactions. Combining these results with those of the previous section, we suggest that Ca^2+^ binding reshapes the sorting signal binding groove in a way that optimal Van der Waals contacts with the peptide are possible and non-polar solvent accessible surface area is diminished upon peptide binding.

Finally, we will briefly discuss two theoretical aspects important for our analysis and the final mechanistic conclusions drawn, namely dependency of the results on the conformational space sampled and the force field applied.

First, in terms of conformational sampling, the capacity of conventional molecular dynamics is often limited by the long-time scale of the studied conformational changes that motivates the introduction of enhanced sampling techniques to circumvent these limitations. However, the conformational sampling capacities of conventional MD and enhanced techniques are comparable if the simulation is long enough to reach the convergence limit of the process of interest. If this is the case, the advantage of conventional MD is that an intact, unbiased continuous dynamic trajectory is obtained, featuring a collection of consecutive conformations linked to each other only by the dynamical behavior of the protein. This is of special interest if the motion cannot be defined easily by any collective variables, as it is the case for the dynamics of intrinsically disordered regions, as studied in this context. In such cases unbiased MD simulations can enlighten mechanistic aspects of the protein’s dynamical behavior [58, 59], which cannot be obtained by conformational sampling based enhanced simulation techniques. In our case comparison of the probability distributions of crucial structural features as d_pep_ and d_Ca_ obtained by the here presented long-term MD simulations with the results of previous enhanced sampling-based studies showed that the conformational space covered during our simulations is at least as comprehensive as in the previous studies.

During our long-term simulations, we detected distinct stable conformations at the beginning and the end of our trajectory, which are connected via several intermediates. We show that the major events that trigger the formation of these intermediates are alterations in the secondary structure content of the disordered loop. Here one should note that, as shown in extensive reviews [60–65], observed changes in the secondary structure of proteins and their stabilities within an MD simulation are force field dependent. Thus, the α-helix formation in the disordered loop might be over or underestimated depending on our choice of force field. However, we did not observe one isolated short-lived folding event of a single helix, but several subsequent folding and unfolding events of different helical regions located in different regions of the loop in correlation with a specific movement in the dynamic loop within 6 distinct MD simulations. Hence, the formation of these helices and their lifetimes might be different than their appearance under physiological conditions; but, considering the frequency of the observed folding/unfolding transitions, it is safe to conclude that the overall dynamical changes observed, namely, the formation of helical regions in the disordered loop as well as their impact on the structure and dynamics of the dynamic loop stand on solid basis…

## Conclusions

We performed long-term molecular dynamics simulations investigating the effect of Ca^2+^ binding on the allosteric regulation of Sa-SrtA. Thereby we focused our analysis on two, previously not investigated aspects of Sa-SrtA-substrate binding: namely, the dynamical aspects of the allosteric changes triggered by Ca^2+^ binding and their structural and energetic effect on substrate binding.

We observed that Ca^2+^ binding leads to correlated sequential changes in the two catalytically important loops. But are these changes and motions crucial in terms of enzyme activity or are they arbitrary? According to our binding free energy estimations, relevant intermediate protein conformations extracted from the Ca^2+^ bound simulation of sortase A feature stronger binding affinities towards the substrate peptide compared to the structures obtained from the Ca^2+^ free sortase A simulation. We correlated these stronger binding affinities to an increase in the hydrophobic ligand-protein interactions and to more compact, stable structures of the bound complexes. This result provides a quantitative explanation for the previously suggested hypothesis claiming an accelerating effect of Ca^2+^ binding for sorting signal binding via efficient reorganization of the disordered loop; we show that the presence of Ca^2+^ is necessary for these conformational changes to occur in favor of the enzymatic activity (i.e., sorting signal binding).

**Fig 6:**
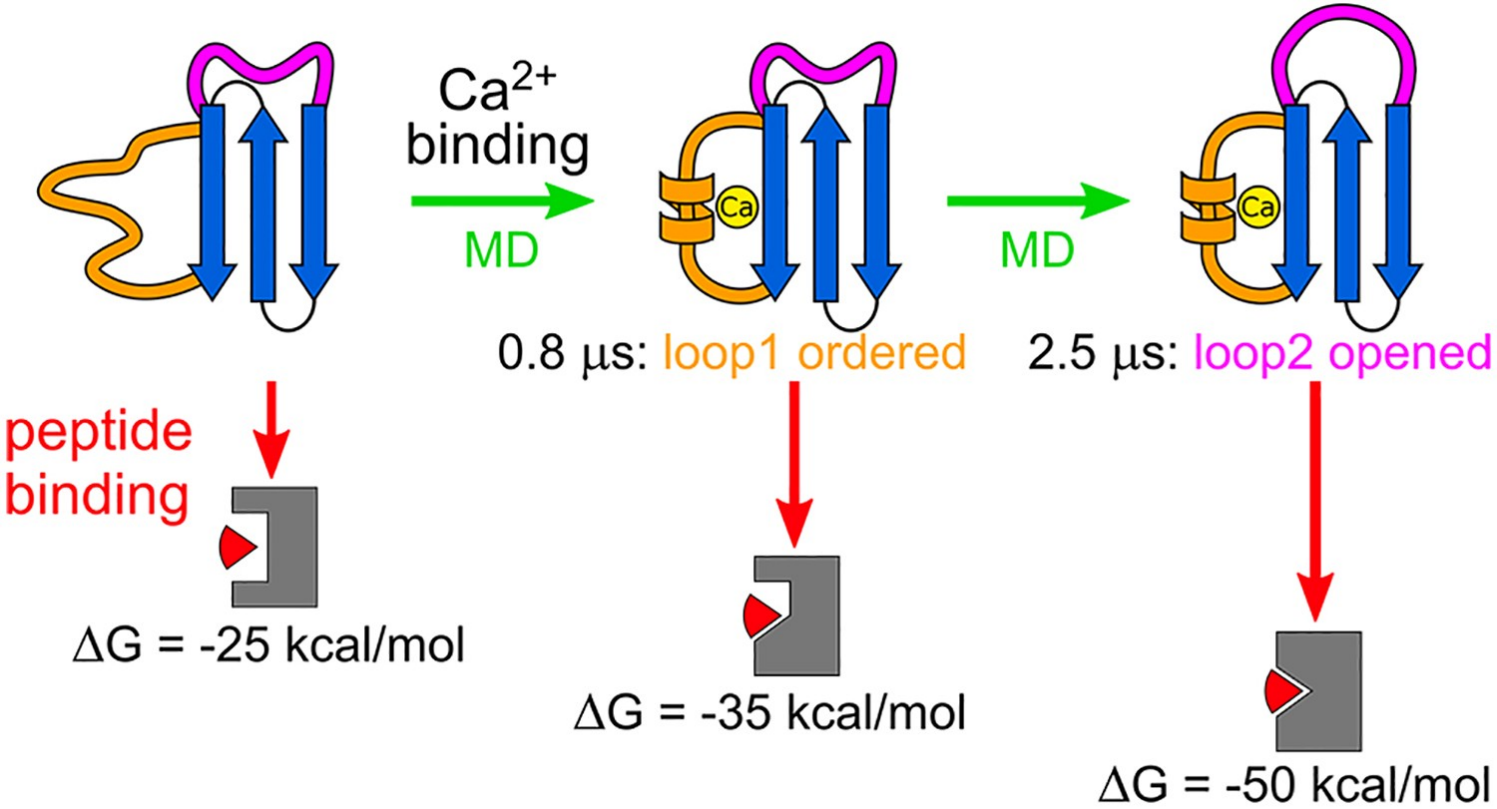
Schematic representation summarizing the effect of Ca^2+^ binding on the sortase structure and on the sortase/sorting signal affinity.

Combining all of these observations, we conclude that Ca^2+^ binding promotes binding of the sorting signal by inducing major changes in the dynamics and conformation of the disordered and dynamic loops located next to the binding groove of the protein. These changes are summarized as follows and schematically represented in Fig 6:

First, the disordered loop, to which Ca^2+^ is directly bound, is rigidified leading to a higher conformational complementary of the binding groove with respect to the sorting signal peptide as shown by both MD simulations and by the experimental Sa-SrtA melting point determination.

Second, the dynamic loop, which is more than 15 Å away from the Ca^2+^ binding site, gradually opens up upon rigidification of the disordered loop, thus allowing the peptide to properly enter the binding groove. Although enzyme activity related loop dynamics has recently been reviewed (triggering/triggered loops) [58], to the best of our knowledge, this complex signal transmission (via alterations in helix structures and several structural intermediates) has never been investigated in comparable detail before to explain the allosteric activation of this intrinsically disordered enzyme.

Third, these correlated structural changes result in tighter bound enzyme-peptide complexes indicating a Ca^2+^-induced conformational selection-driven peptide binding mechanism.

Overall, our molecular dynamic simulations showed that binding of Ca^2+^ initiates the allosteric regulation of sortase A-peptide binding. This regulation consists of sequential conformational changes, which lead to intermediate binding site conformations optimal for protein-peptide assembly. Therefore, our results reveal that the activation of sortase A through Ca^2+^-binding initiates an allosteric, conformational selection-driven activation mechanism of the enzyme involving disorder-to-order transitions in the intrinsically disordered regions of the protein.

We currently perform further studies to elucidate if this correlated dynamics is also related with the specificity of the enzyme towards the well-conserved LPXTG sequence of the sorting signal.

## Supporting information

S1 Fig RMSD time series of the two replica simulations of SrtA-Ca and SrtA-noCa. a-b) SrtA-Ca. c-d) SrtA-noCa. The black, red, magenta, and blue colors represent the deviations of the backbone atoms of the protein, residues assembling the eight-stranded β-barrel (β sheets), the disordered loop (loop-dis), and the dynamic loop (loop-dyn), respectively.
(TIF)

S2 Fig Structural analysis of the disordered and dynamic loops in the replica simulations of SrtA-Ca. a, b) Variations of the secondary structure in the disordered loop. c, d) Fluctuations of the distances characterizing the motion of the dynamic loop.
(TIF)

S3 Fig Structural analysis of the disordered and dynamic loops in the simulation SrtA-noCa. a) Variations of the secondary structure in the disordered loop. b) Fluctuations of the distances characterizing the motion of the dynamic loop. c) Representative structures of the different loop conformations. d) Representative structure of the protein showing the twisted orientation of the dynamic loop.
(TIF)

S4 Fig Structural analysis of the disordered and dynamic loops in the replica simulations of SrtA-noCa. a, b) Variations of the secondary structure in the disordered loop. c, d) Fluctuations of the distances characterizing the motion of the dynamic loop.
(TIF)

S5 Fig Temperature flow measurments of Sa-SrtA, with and without Ca^2+^. Three independent measuremts were performed for each sample w/o 10 mM Ca^2+^. The probes were heated from 20 to 90 °C by a rate of 90 °C/h.
(TIF)

S6 Fig Time series of the clustering analysis. a) SrtA-Ca. b) SrtA-noCa. The most populated cluster is numbered as cluster 0.
(TIF)

S7 Fig Representative structures of the clusters obtained from the SrtA-Ca simulation. (TIF)
