# Supplementary figures and images for: Ca2+ binding induced sequential allosteric activation of sortase A: An example for ion-triggered conformational selection
Source: PLoS One. 2018 Oct 15;13(10):e0205057. doi: 10.1371/journal.pone.0205057 (PMC6188747; doi:10.1371/journal.pone.0205057)

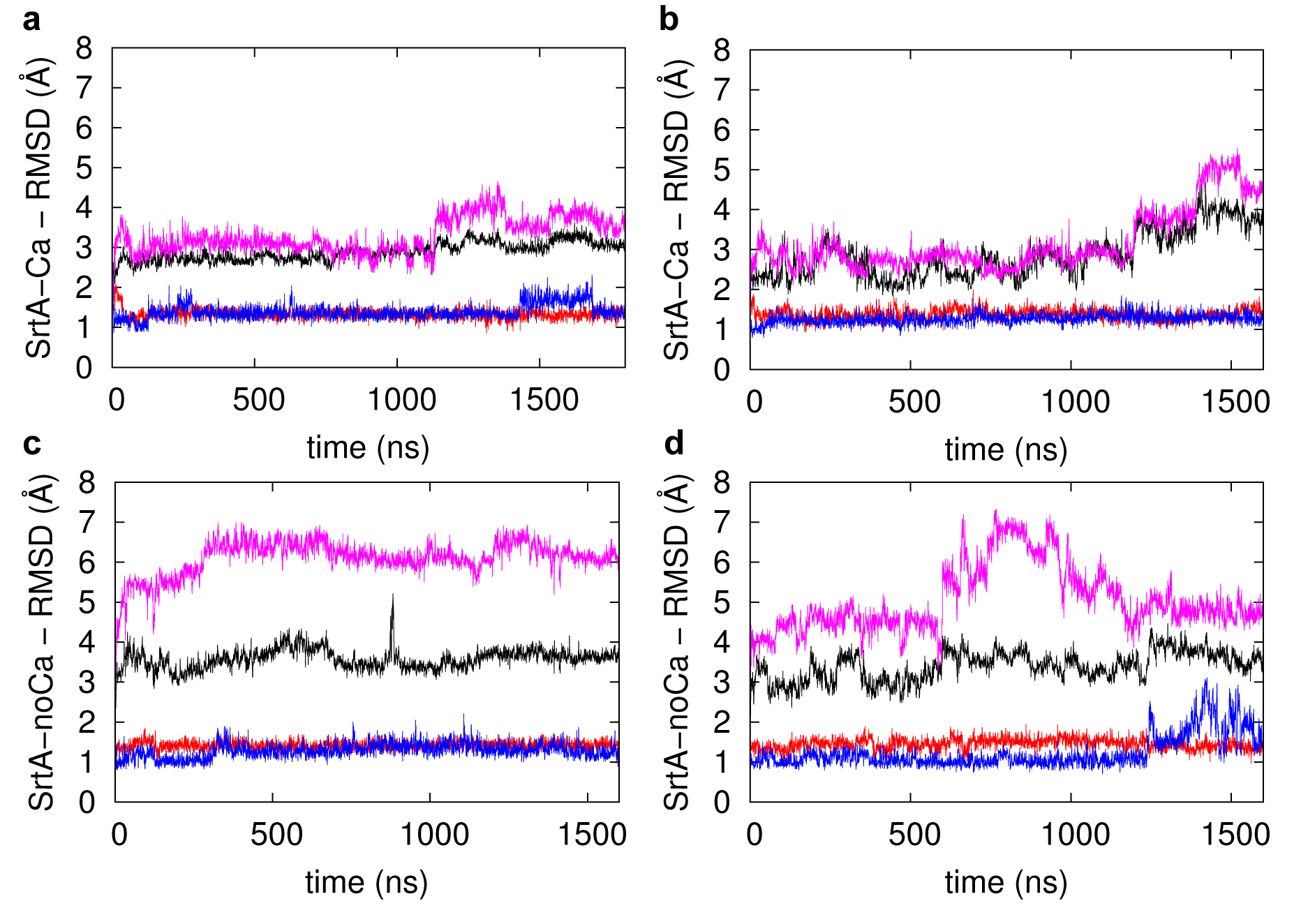

Supplement: S1 Fig — a-b) SrtA-Ca. c-d) SrtA-noCa. The black, red, magenta, and blue colors represent the deviations of the backbone atoms of the protein, residues assembling the eight-stranded β-barrel (β sheets), the disordered loop (loop-dis), and the dynamic loop (loop-dyn), respectively. (TIF) [file pone.0205057.s001.tif]

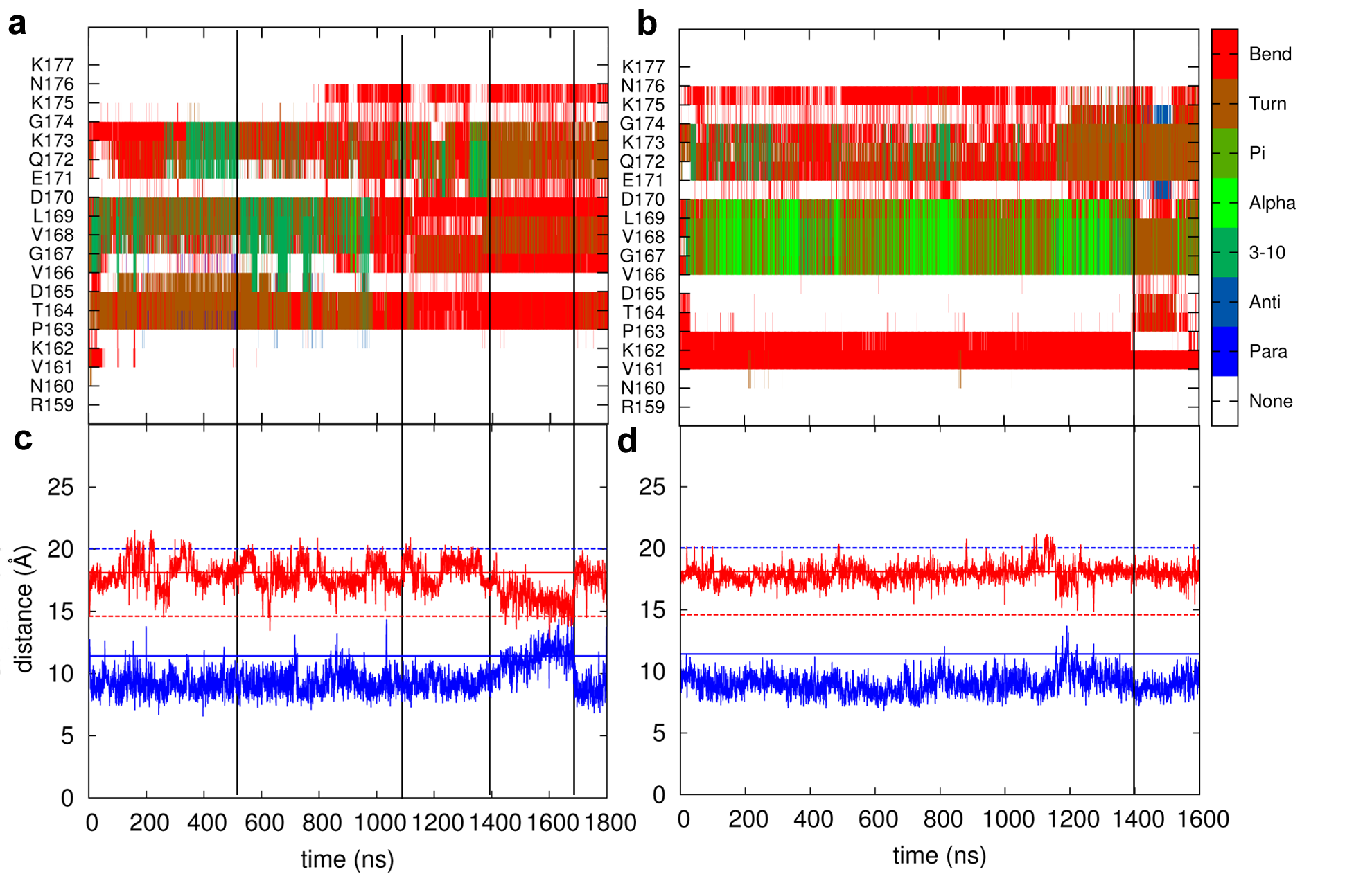

Supplement: S2 Fig — a, b) Variations of the secondary structure in the disordered loop. c, d) Fluctuations of the distances characterizing the motion of the dynamic loop. (TIF) [file pone.0205057.s002.tif]

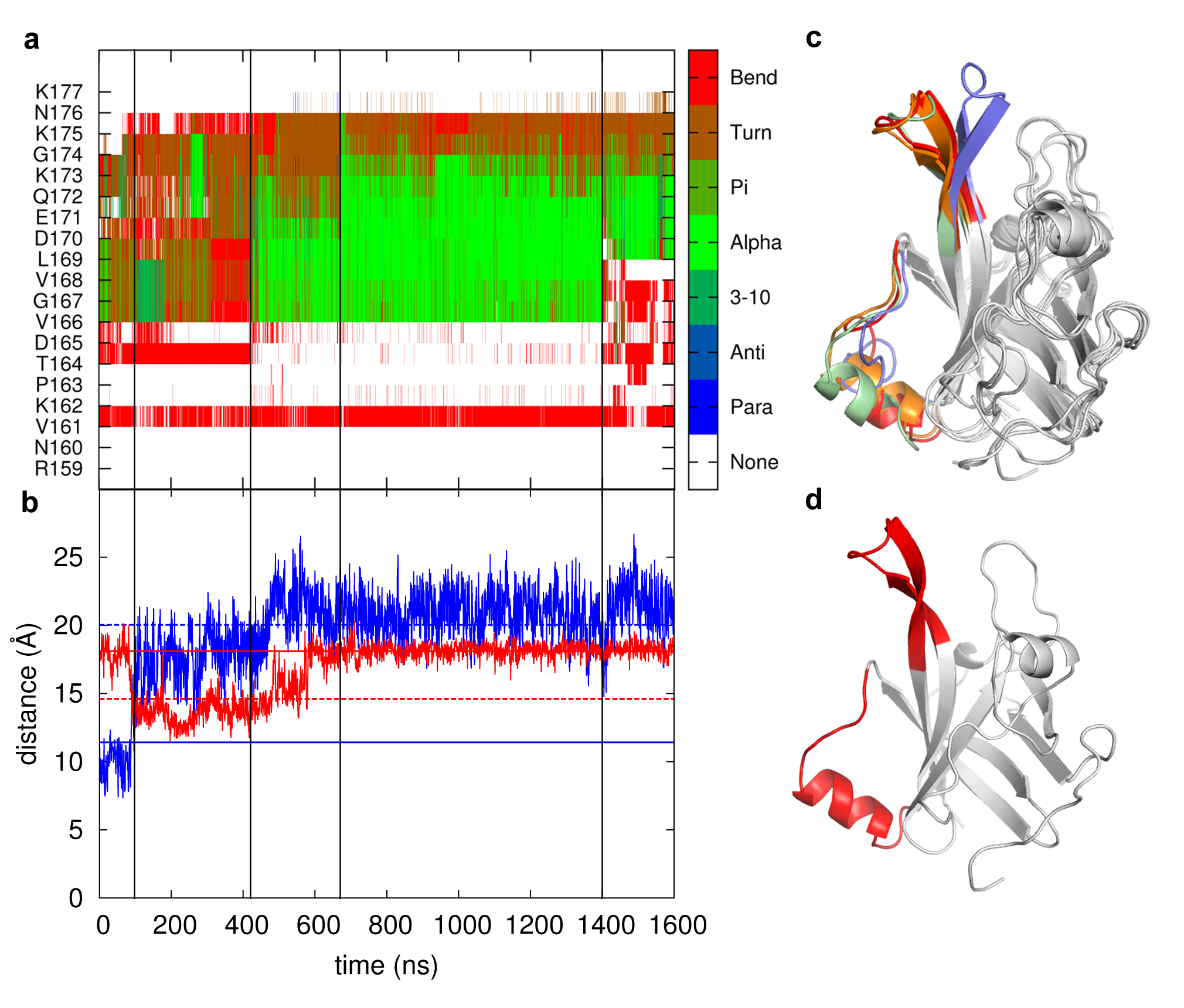

Supplement: S3 Fig — a) Variations of the secondary structure in the disordered loop. b) Fluctuations of the distances characterizing the motion of the dynamic loop. c) Representative structures of the different loop conformations. d) Representative structure of the protein showing the twisted orientation of the dynamic loop. (TIF) [file pone.0205057.s003.tif]

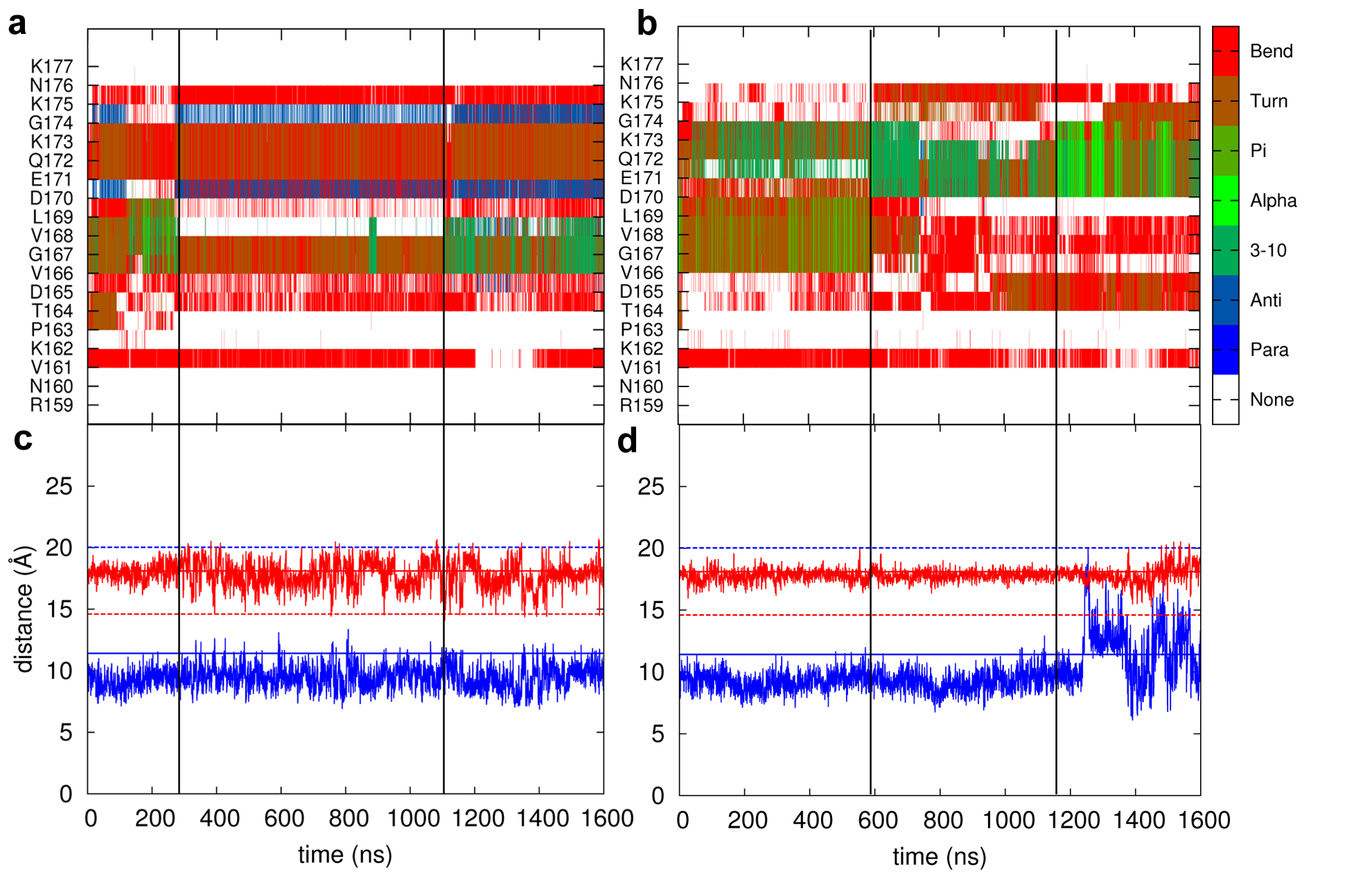

Supplement: S4 Fig — a, b) Variations of the secondary structure in the disordered loop. c, d) Fluctuations of the distances characterizing the motion of the dynamic loop. (TIF) [file pone.0205057.s004.tif]

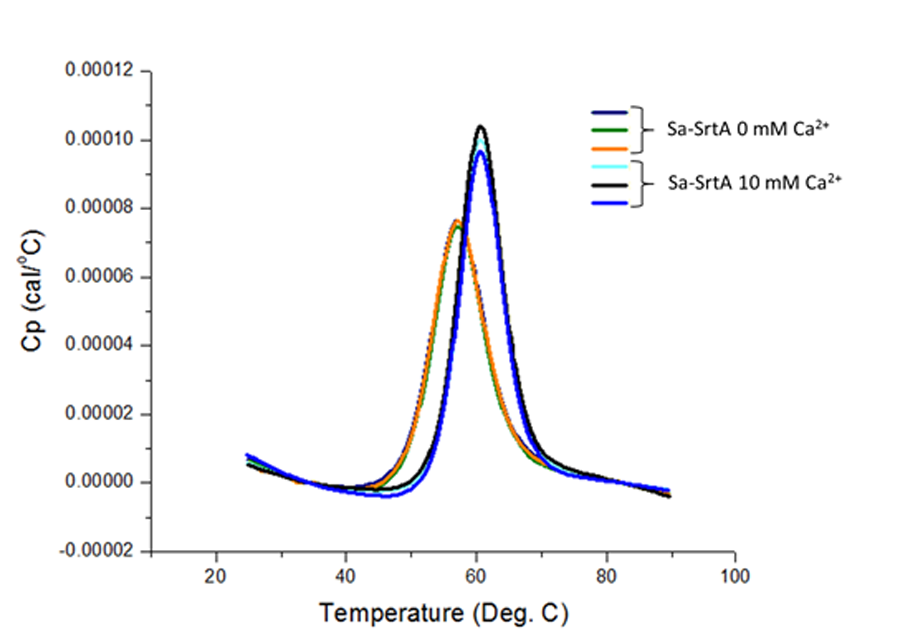

Supplement: S5 Fig — Three independent measuremts were performed for each sample w/o 10 mM Ca2+. The probes were heated from 20 to 90 °C by a rate of 90 °C/h. (TIF) [file pone.0205057.s005.tif]

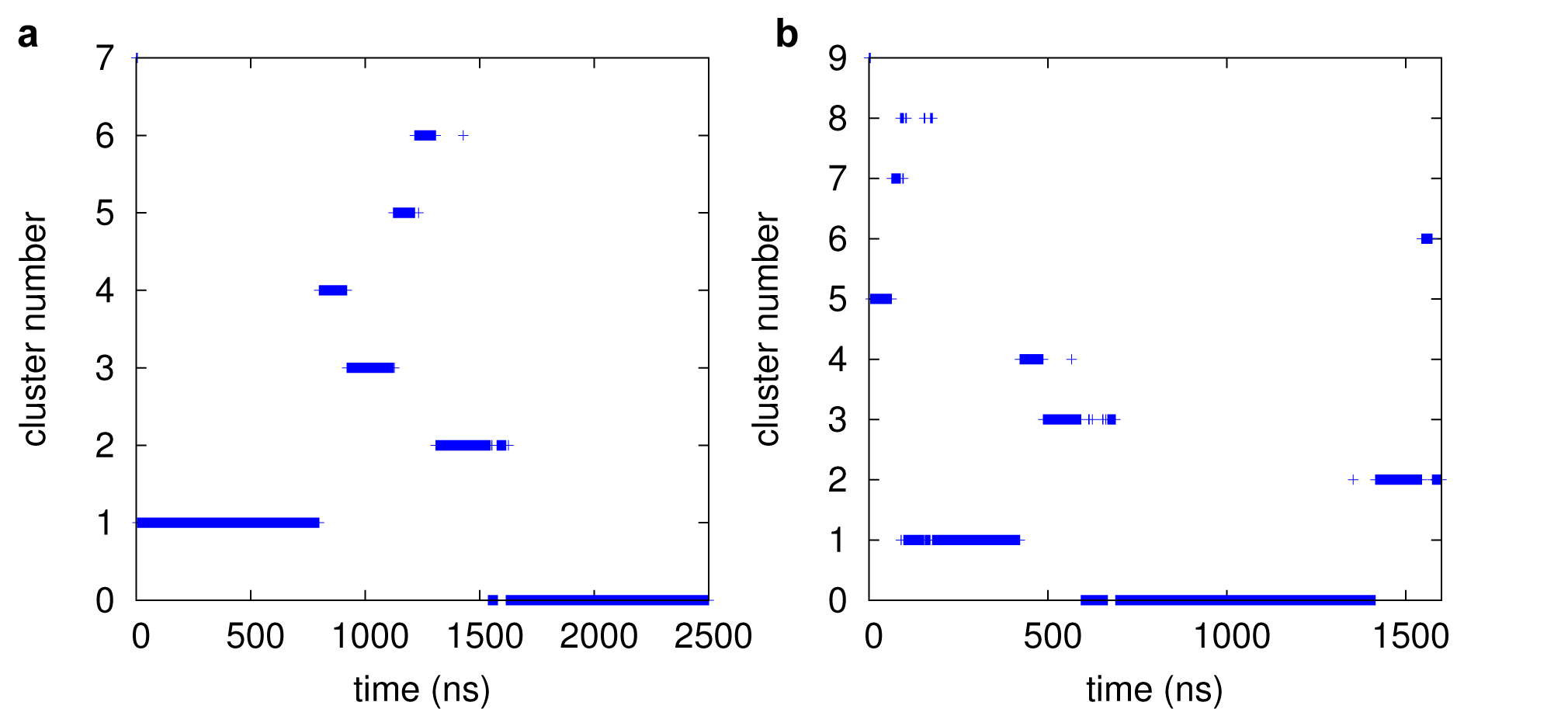

Supplement: S6 Fig — a) SrtA-Ca. b) SrtA-noCa. The most populated cluster is numbered as cluster 0. (TIF) [file pone.0205057.s006.tif]

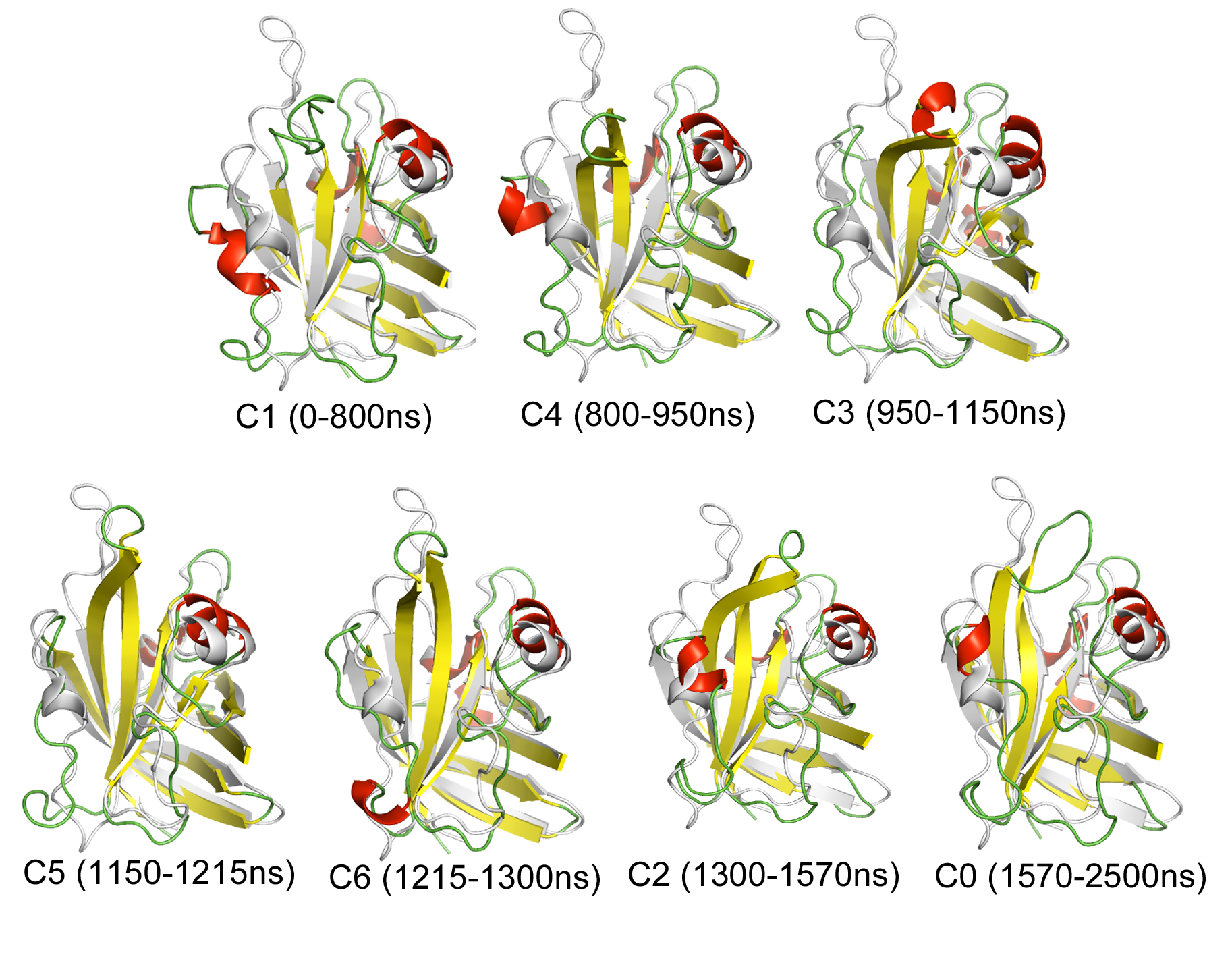

Supplement: S7 Fig — (TIF) [file pone.0205057.s007.tif]
